# The CCAAT/Enhancer-Binding Protein Beta-2 Isoform (CEBPβ-2) Upregulates Galectin-7 Expression in Human Breast Cancer Cells

**DOI:** 10.1371/journal.pone.0095087

**Published:** 2014-05-02

**Authors:** Carole G. Campion, Marilyne Labrie, Andrée-Anne Grosset, Yves St-Pierre

**Affiliations:** INRS-Institut Armand-Frappier, Laval, Québec, Canada; University of Illinois at Chicago, United States of America

## Abstract

*Galectin-7* is considered a gene under the control of p53. However, elevated expression of galectin-7 has been reported in several forms of cancer harboring an inactive p53 pathway. This is especially true for breast cancer where galectin-7 expression is readily expressed in a high proportion in basal-like breast cancer tissues, conferring cancer cells with increased resistance to cell death and metastatic properties. These observations suggest that other transcription factors are capable of inducing *galectin-7* expression. In the present work, we have examined the role of CCAAT/enhancer-binding protein beta (C/EBPβ) in inducing expression of galectin-7. C/EBP proteins have been shown to contribute to breast cancer by upregulating pro-metastatic genes. We paid particular attention to C/EBPβ-2 (also known as LAP2), the most transcriptionally active of the C/EBPβ isoforms. Our results showed that ectopic expression of C/EBPβ-2 in human breast cancer cells was sufficient to induce expression of galectin-7 at both the mRNA and protein levels. *In silico* analysis further revealed the presence of an established CEBP element in the *galectin-7* promoter. Mutation of this binding site abolished the transcriptional activity of the *galectin-7* promoter. Chromatin immunoprecipitation analysis confirmed that C/EBPβ-2 binds to the endogenous *galectin-7* promoter. Analysis of galectin-7 protein expression in normal epithelia and in breast carcinoma by immunohistochemistry further showed the expression pattern of C/EBPβ closely micmicked that of galectin-7, most notably in mammary myoepithelial cells and basal-like breast cancer where galectin-7 is preferentially expressed. Taken together, our findings suggest that C/EBPβ is an important mediator of *galectin-7* gene activation in breast cancer cells and highlight the different transcriptional mechanisms controlling galectin-7 in cancer cells.

## Introduction

Galectins constitute a family of lectins defined by shared consensus amino acid sequences and affinity for β-galactose-containing oligosaccharides [1]. In mammals, the distribution of galectins is tissue-specific and their expression is developmentally regulated [1], [2]. They play an important role in several physiological processes, including embryonic development, wound healing, apoptosis, intercellular adhesion, cell migration, and immune response. They are also involved in a number of pathological conditions, including infectious diseases and cancer. Most of our knowledge on galectins has been obtained while studying galectin-1 and galectin-3. In contrast, galectin-7 remains an unfamiliar member of the galectin family. This galectin was initially described as a marker of differentiation of stratified epithelia by Magnaldo and colleagues [3]. Functionally, galectin-7 has been shown to be associated with UVB-induced apoptosis in epidermis since sunburn/apoptotic keratinocytes express abnormally high levels of galectin-7 [4]. In fact, galectin-7 has been considered an apoptosis regulator in many cell systems since its discovery by the group of Bert Vogelstein as one of the 14 transcripts out of 7,202 induced in colorectal cancer cells by the expression of p53, whose major function is to control apoptosis [5].

In sharp contrast to the intuitively expected negative roles played by galectin-7 in tumor development, a study by Lu *et al*. reported that galectin-7 was overexpressed in tumors induced in an experimental model of chemically induced mammary carcinomas [6]. This provided the first indication that galectin-7 expression could be modulated in breast cancer. We and others have since shown that galectin-7 is also expressed in breast cancer tissues in humans. For example, genomic profiling data reported by Perou *et al*., who provided a molecular portrait of human breast tumors from 42 individuals, has revealed that galectin-7 was highly expressed in estrogen receptor (ER)-negative tumors [7]. Subsequent microarray studies examining normal breast luminal and myoepithelial cells by Jones and his colleagues identified *galectin-7* as a myoepithelial-specific gene [8]. We have since confirmed the specific expression pattern of galectin-7 in breast cancer tissues using tissue microarrays (TMAs) constructed from samples obtained from normal individuals and in patients with breast carcinomas [9]. Using preclinical mouse models, we have further shown that high levels of galectin-7 expression in breast cancer cells increase their ability to metastasize to lungs and bones [9].

While a clear picture of the function of galectin-7 in breast cancer is emerging, very little is known about the molecular mechanisms regulating galectin-7 expression in human breast cancer cells. Among the transcription factors that could possibly regulate galectin-7 is CCAAT/enhancer binding protein beta (C/EBPβ). Members of the C/EBPβ family have been shown to contribute to tumor progression by controlling the expression of genes involved in invasion, cellular proliferation, survival and apoptosis [10]–[13]. In mammary tissues, this factor has been shown to be critical for normal growth and differentiation of the mammary gland [14]–[16]. It also contributes to malignant conversion of the human breast [12]. Of the three C/EBPβ isoforms, a particular attention has been paid to C/EBPβ-2 because overexpression of this isoform induces epithelial-mesenchymal transition [17]. A significant increase in C/EBPβ is observed in estrogen and progesterone-receptor-negative breast cancer as compared to tumors positive for these receptors. Increased C/EBPβ levels also correlate with metastatic breast cancer and a high tumor grade [12]. In the present work, we have examined whether C/EBPβ regulates expression of galectin-7 in breast cancer cells.

## Materials and Methods

### Cell lines and reagents

Breast cancer cell lines were a generous gift from Dr. P. Siegel (the Goodman Cancer Centre, McGill University, Montreal, Qc) [18]. Immortalized human keratinocytes (HaCaT) were provided by Dr. T. Magnaldo (Université de Nice) [4]. All cell lines were maintained in Dulbecco's modified Eagle's medium. Culture media were supplemented with 10% [v/v] fetal bovine serum, 2 mM L-glutamine, 10 mM HEPES buffer, 1 mM non-essential amino acids and 1 mM Sodium Pyruvate. All cell culture products were purchased from Life Technologies (Burlington, ON, Canada).

### Transient transfection and luciferase assay

Vectors encoding human C/EBPα or β (SC303472 and SC319561; Origene, Burlington, ON) and vectors encoding C/EBPβ-2 and C/EBPβ-3 (No. 15738 and No. 15737; Addgene, Cambridge, MA) were obtained commercially. The cDNA encoding the human galectin-7 (provided by Dr. T. Magnaldo) was cloned in the srα eukaryotic expression vector (kind gift of Dr. François Denis) using SpeI and BamHI restriction sites. Cells were transfected using the Lipofectamine 2000 reagent (Life Technologies) according to the manufacturer's protocol. For reporter assays, NF-κB or a C/EBP luciferase reporter vectors (Cat. No. 219078 and 240112 respectively; Stratagene, Santa Clara, CA) were used. The (empty) pCMV5 vector was used as a control. Transfection efficiency was measured using the pCMV/β-gal reporter vector (Promega, Madison, WI). Luciferase activity was measured using the Luciferase Assay System protocol (Promega) and a luminometer (Lumat LB 9507, Berthold). β-galactosidase activity was detected using a colorimetric enzyme assay using the Luminescent β-Galactosidase Detection Kit II according to the manufacturer's instructions (Clontech Laboratories, Mountain View, CA). Luciferase expression levels were normalized to the levels of β-galactosidase expression.

### Western blot analysis

Cells were solubilized in radioimmunoprecipitation assay (RIPA) lysis buffer (Thermo Fisher Scientific, Ottawa, ON) containing a cocktail of protease inhibitors (Roche, Laval, QC). Equal amounts of protein (25 µg) were separated on SDS-PAGE and transferred onto nitrocellulose membranes (Bio-Rad Laboratories, Mississauga, ON). The membranes were first blocked with 5% (v/v) milk in PBS/0.05% Tween 20 for 1 h and subsequently blotted overnight at 4°C with primary antibodies: rabbit anti-C/EBPβ polyclonal antibody (1∶1000; sc-150 (C-19) Santa-Cruz Biotechnology, Santa Cruz, CA) and mouse anti-β-actin monoclonal antibody (1∶20000; Sigma, St.Louis, MO, USA). Secondary antibodies consisted of horseradish peroxydase conjugated anti-rabbit or anti-mouse (GE Healthcare, Mississauga, ON). The immunoblots were developed using ECL detection reagent (GE Healthcare).

### Confocal microscopy

Cells were cultured onto glass coverslips to semi-confluency. After 24 h of transfection with vector encoding C/EBPβ-2, cells were washed with cold PBS, fixed with 3% (v/v) paraformaldehyde in PBS and permeabilized with 0.1% Triton X-100 in PBS and blocked with 1% (v/v) BSA in PBS (PBA) for 30 min. Cells were first incubated overnight at 4°C with a goat anti-human galectin-7 polyclonal antibody (1∶100; R&D Systems, Minneapolis, MN) with PBA. After several washes, cells were incubated with an Alexa Fluor 488-conjugated donkey anti-goat IgG (1∶500; Life Technologies) for 1 h at room temperature. Samples were mounted using the ProLong gold antifade reagent together with 4′-6-diamino-2-phenylindole (DAPI) (Life Technologies) on glass slides and visualized using a Zeiss LSM780 laser scanning microscope (Carl Zeiss Microimaging, Thornwood, NY).

### RNA Isolation and RT-PCR

Total cellular RNA was isolated from cells using the TRIzol reagent (Life Technologies) according to the manufacturer's instructions. First-strand cDNA was prepared from 2 µg of cellular RNA in a total reaction volume of 20 µL using the reverse transcriptase Omniscript (QIAGEN, Mississauga, ON, Canada). After reverse transcription, human *galectin-7* (gene ID 3963, sense primer: 5′- ACC AAC CCG GTC CCA G -3′ and antisense primer: 5′- GCG GGC TAA CGC TTT ATT TGC -3′) and *GAPDH* (gene ID 2597, sense primer: 5′- CGG AGT CAA CGG ATT TGG TCG TAT-3′ and antisense primer: 5′-CAG AAG TGG TGG TAC CTC TTC CGA -3′) cDNAs were amplified using the following conditions: 94°C for 3 min, followed by 35 cycles of the following: 94°C for 1 min, 60°C for 1 min, and 72°C for 1 min, followed by a final extension step at 72°C for 10 min. PCR was performed in a thermal cycler (MJ Research, Watertown, MA). The amplified products were analyzed by electrophoresis using 1.5% (w/v) agarose gels and SYBR Safe DNA gel (Invitrogen) staining and UV illumination.

### Chromatin immunoprecipitation (ChIP)

ChIP assays were performed using the EZ-Chromatin-Immunoprecipitation Assay Kit (Millipore, Billerica, MA). For this purpose, MCF-7 cells were grown overnight in 100-mm dishes to ∼60–70% confluency and then transfected with either the empty pCMV5 control vector or vectors encoding C/EBPβ-2 or C/EBPβ-3 using Lipofectamine 2000 reagent. Cells were fixed in 1% (v/v) paraformaldehyde. Nuclei were isolated, sonicated, and pre-cleaned with protein G agarose/salmon sperm DNA. The pre-cleaned chromatin solution was either set aside as input DNA or incubated with anti-NF-κB/p50 (Cat. No. 06-886; Millipore) or anti-C/EBPβ (C-19, sc-150; Santa-Cruz Biotechnology) on a rotation platform at 4°C overnight. Input DNA and mouse IgG-pulled DNA were used as controls for all the experiments. After reversal of the cross-linking, DNA was purified from the immune complex and amplified using PCR primers specific for the *galectin-7* promoter region encompassing +1 to −200 bp: sense: 5′-CCT GGG TGA TGG GGG GAT CAGG -3′ and antisense: 5′-CCA TGT CCG TGA GTG CTC CAG GG-3′. The samples were incubated for 3 min at 94°C, followed by 40 cycles as follows: 1 min at 94°C, 1 min at 62°C, and 1 min at 72°C, with a final extension at 72°C for 10 min.

### Scratch wound healing assay

Confluent monolayers were obtained by seeding 3×10^5^ cells onto 6-well glass bottom culture plates (MatTek Corporation, Ashland, MA). Cells were transfected with vectors encoding specific cDNA using the Lipofectamine 2000 reagent (Life Technologies) according to the manufacturer's protocol. After 24 h, a scratch with a pipet tip was made in the cell monolayer, followed by washing with PBS to remove cell debris. The plates were moved to an incubator and migration was visualized with a Carl Zeiss LSM780 confocal microscope (Carl Zeiss). Images were captured every 10 min for 2 h. For each cell type, the movement of 30 or 60 different cells was measured. Cell movement was analyzed using the Image J plugins manual tracking and chemotaxis tool.

### Statistical analysis

Statistical significance was carried out using an unpaired Student's t-test. Results were considered statistically significant at P≤0.05.

## Results

### C/EBPβ-2 induces galectin-7 expression in breast cancer cell lines

**Figure 1:**
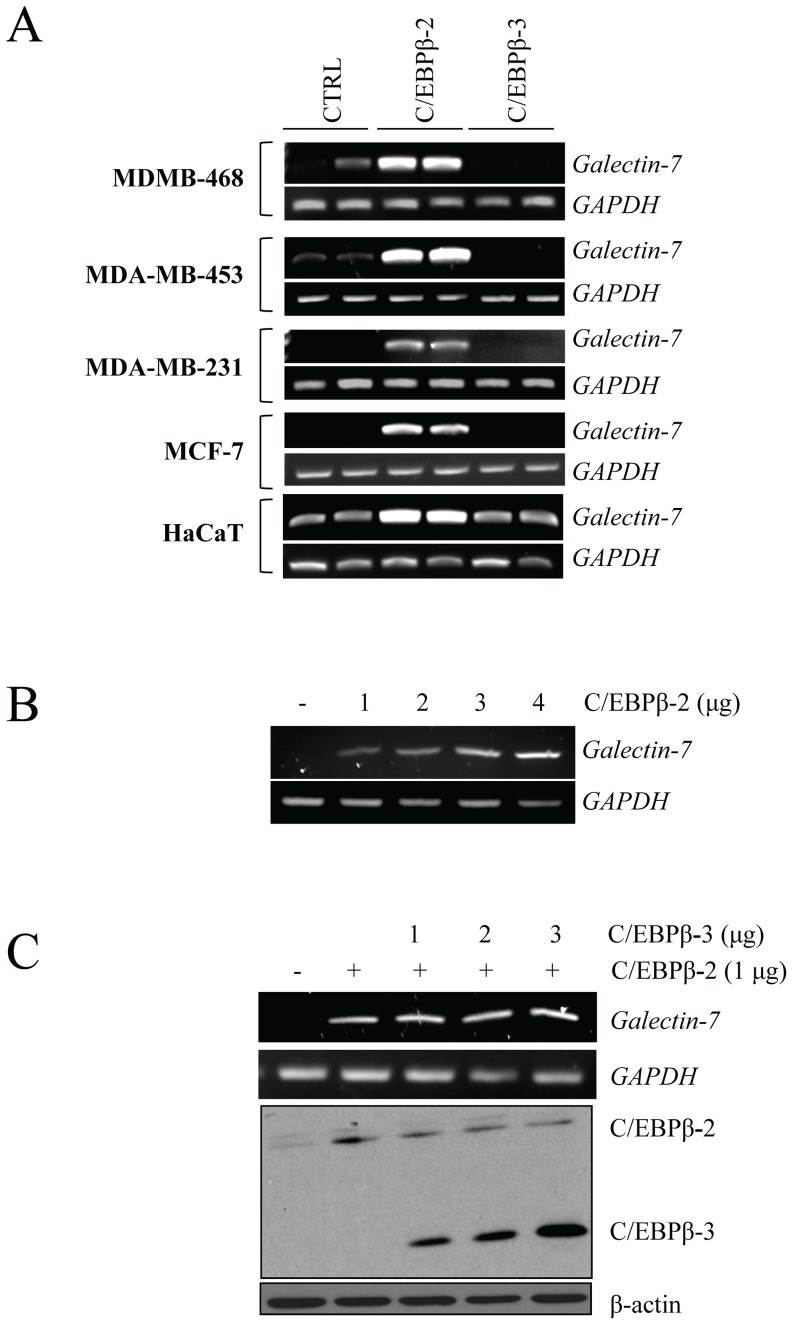
C/EBPβ-2 induces *galectin-7* mRNA levels in breast cancer cell lines. (**A**) RT-PCR analysis showing increased expression of *galectin-7* in human breast cancer cells after transfection with an expression vector encoding C/EBPβ-2. The two lanes represent two different samples. No such increase was observed in cells transfected with an expression vector encoding C/EBPβ-3. Similar results were obtained with HaCaT cells, a keratinocyte cell line which constitutively expresses *galectin-7*. An empty pCMV5 vector was used as transfection control (CTRL) and GAPDH was used as loading control. (**B**) RT-PCR analyses showing expression of *galectin-7* mRNA levels in MCF-7 cells after transfection with increasing doses of an expression vector encoding C/EBPβ-2. GAPDH was used as loading control. (**C**) RT-PCR analysis of MCF-7 cells co-transfected with vectors encoding C/EBPβ-2 and C/EBPβ-3. Below, control Western blot analysis showing expression of C/EBPβ-2 and C/EBPβ-3 after transfection. β-actin was used as loading control.

**Figure 2:**
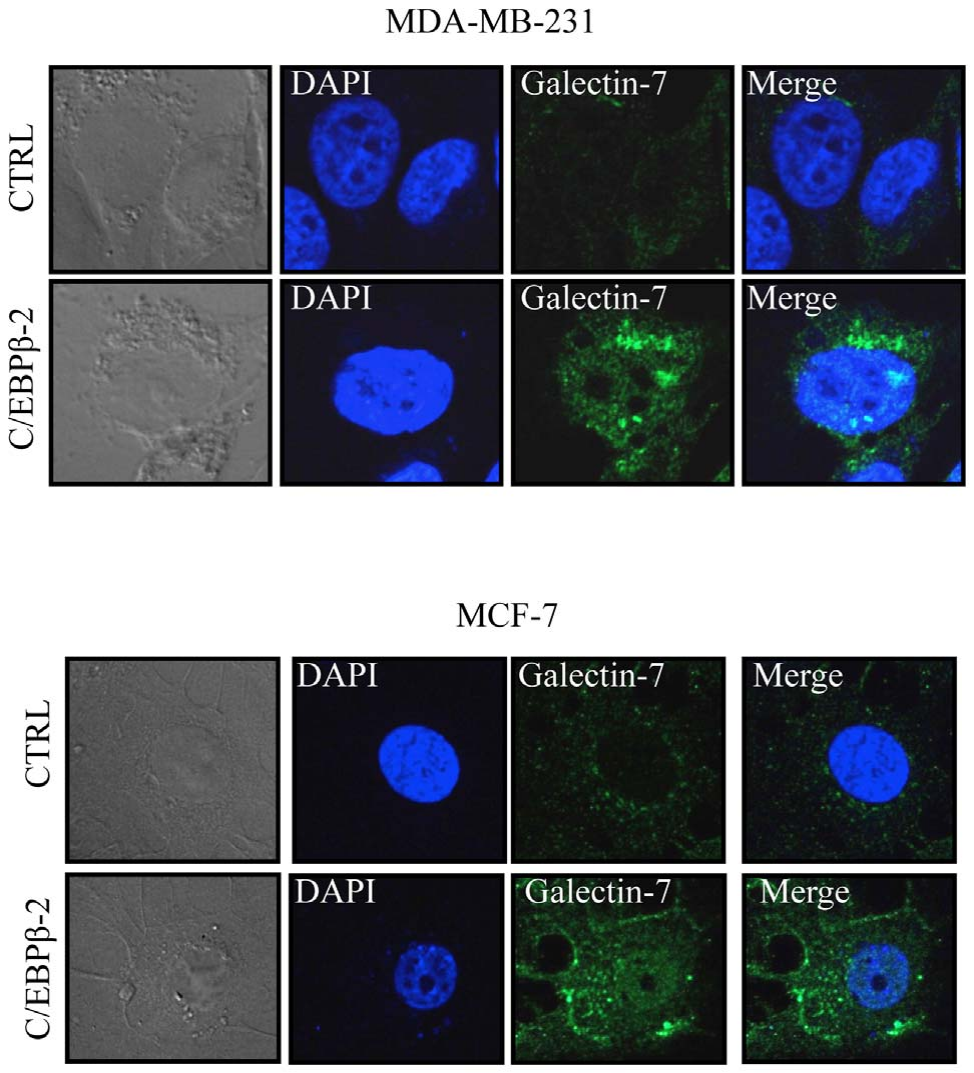
Protein expression of galectin-7 in breast cancer cell lines. MCF-7 or MDA-MB-231 cells were transfected with an expression vector encoding C/EBPβ-2 before cell fixation and permeabilization. A goat anti-human galectin-7 polyclonal antibody was used in combination with an Alexa Fluor 488-conjugated donkey anti-goat IgG to detect endogenous galectin-7 (green). Nuclei were stained with DAPI (blue).

We first set out to determine if the C/EBPβ-2 isoform could induce *galectin-7* gene expression in human breast cancer cell lines. Using semi-quantitative RT-PCR, we found that *galectin-7* mRNA increased in all cell lines tested following transfection of a vector encoding the C/EBPβ-2 isoform (**Figure 1A**). This expression by C/EBPβ-2 was independent of the p53 status of the cells. It was observed in cells expressing a wild-type form of p53, such as MCF-7, or in cells harboring an inactive p53 pathway, such as the p53^null^ MDA-MB-453 or MDA-MB-231, which express the p53^R280K^ mutant protein. It was also found in HaCaT cells, which express the transcriptionally inactive *TP53* ^H179Y/R282W^ alleles. In all cases, *de novo* expression of *galectin-7* was not induced following transfection of a vector encoding C/EBPβ-3 (also known as LIP). This increased expression of galectin-7 by C/EBPβ-2 was also dose-dependent (**Figure 1B**). C/EBPβ-3, which lacks the N-terminal transactivation domains but represses the transcription activity of other C/EBPs by competing for C/EBP consensus binding sites or by forming inactive heterodimers with other C/EBPs [12], [19], did not repress the constitutive expression of *galectin-7* in MDA-MB-468 or HaCaT cells, nor did it repress C/EBPβ-2-induced *galectin-7* (**Figure 1C**). The ability of C/EBPβ-2 to induce galectin-7 was confirmed at the protein level by confocal microscopy in both MDA-MB-231 and MCF-7 cells (**Figure 2**). Taken together, these results indicate that increased expression of C/EBPβ-2 is sufficient to induce galectin-7 in breast cancer cell lines and possibly other types of cells.

### A C/EBPβ consensus site in human *galectin-7* promoter

**Figure 3:**
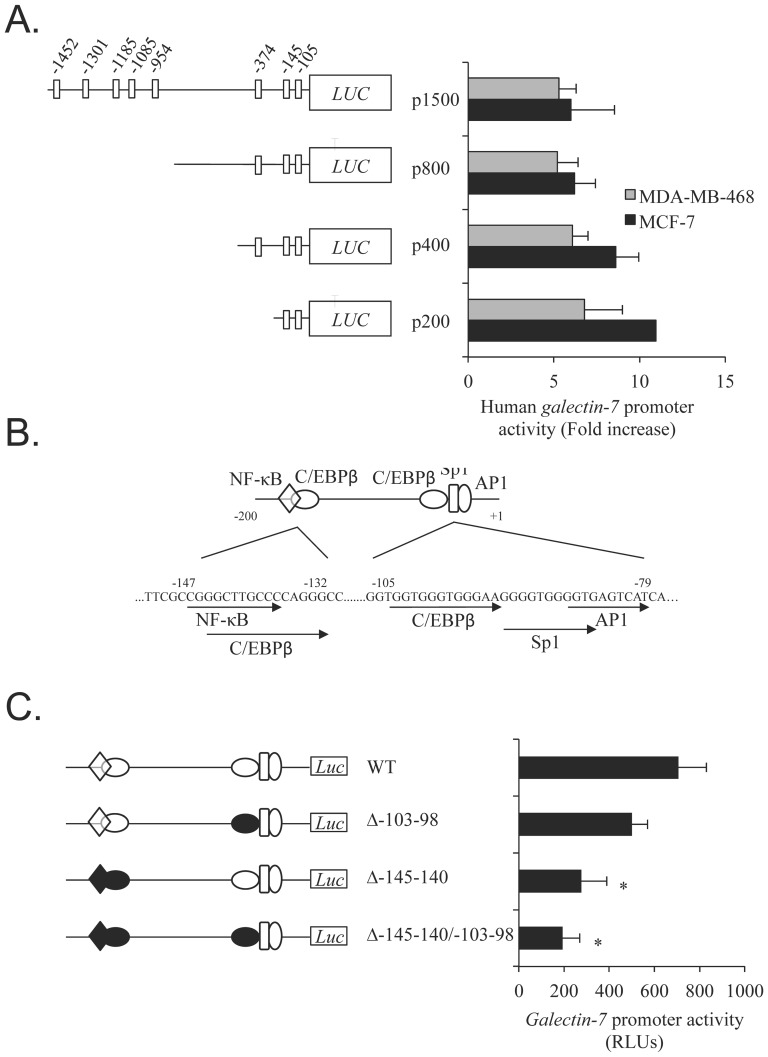
C/EBPβ consensus binding site in *galectin-7* promoter. (**A**) Schematic representation of C/EBP binding sites within the 5′ flanking region of the human *galectin-7* gene. A **s**eries of 5′ deletion constructs of the 1500 bp *galectin-7* promoter region was generated and cloned into the pGL3 Basic luciferase reporter vector. The resulting plasmids were transfected in MCF-7 and MDA-MB-468 cells. Locations of the putative C/EBP binding sites in the promoter, as determined using the TFsearch computational tool, are shown as empty boxes. (**B**) Sequence analysis of the C/EBP binding sites located at positions -105-98 and the -145-140. (**C**) Mutated constructs of the C/EBPβ binding sites on the 200 bp *galectin-7* promoter region were generated and cloned into the pGL3 Basic luciferase reporter vector. The resulting plasmids were co-transfected in MCF-7 cells and were compared to the wild-type *p200-galectin-7* promoter. Transfection efficiency was normalized by co-transfection with a β-galactosidase reporter vector.

*In silico* computational analysis of the human *galectin-7* promoter region (using the TFSEARCH program) revealed eight potential C/EBP binding sites within the proximal 1.5 kb 5′ flanking region of the *galectin-7* gene. Using progressive deletion reporter constructs of the 5′ flanking region of the *galectin-7* promoter, we found that deletion of the distal C/EBP binding sites did not modulate the transcriptional activity of the promoter as compared to the transcriptional activity of a reporter construct containing the two promoter-proximal C/EBP binding sites located at positions −105/−93 bp and −147/−132 bp (**Figure 3A**). Mutational analysis showed that mutation (Δ-103-98) of the C/EBPβ binding site located at −105/−93 bp had minimal effect on the transcriptional activity of *galectin-7* promoter compared to the wild-type promoter. On the other hand, disruption of C/EBPβ binding site located at position −147/−132 bp (Δ-145-140) resulted in a 2.5-fold decrease in the promoter activity (**Figure 3B**). A 3.5-fold decrease was observed when both C/EBPβ binding sites were disrupted (Δ-145-140/-103-98). These findings provide additional evidence that C/EBP plays an important role in controlling *galectin-7* promoter activity.

### Expression of galectin-7 and C\EBPβ in epithelial tissues

**Figure 4:**
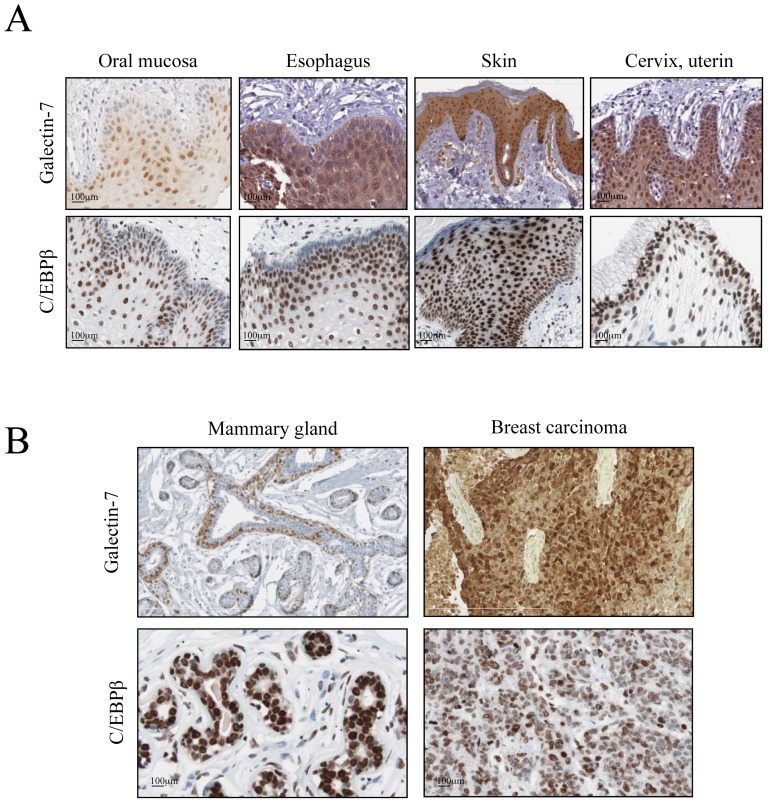
Correlation between expression of galectin-7 and C\EBPβ in epithelial tissues. Immunohistological analysis of galectine-7 and C/EBPβ expression in (**A**) human normal epithelial tissues and (**B**) in normal mammary gland tissue and breast carcinoma. Data were provided by the human protein atlas database (http://www.proteinatlas.org/) and by [9].

**Figure 5:**
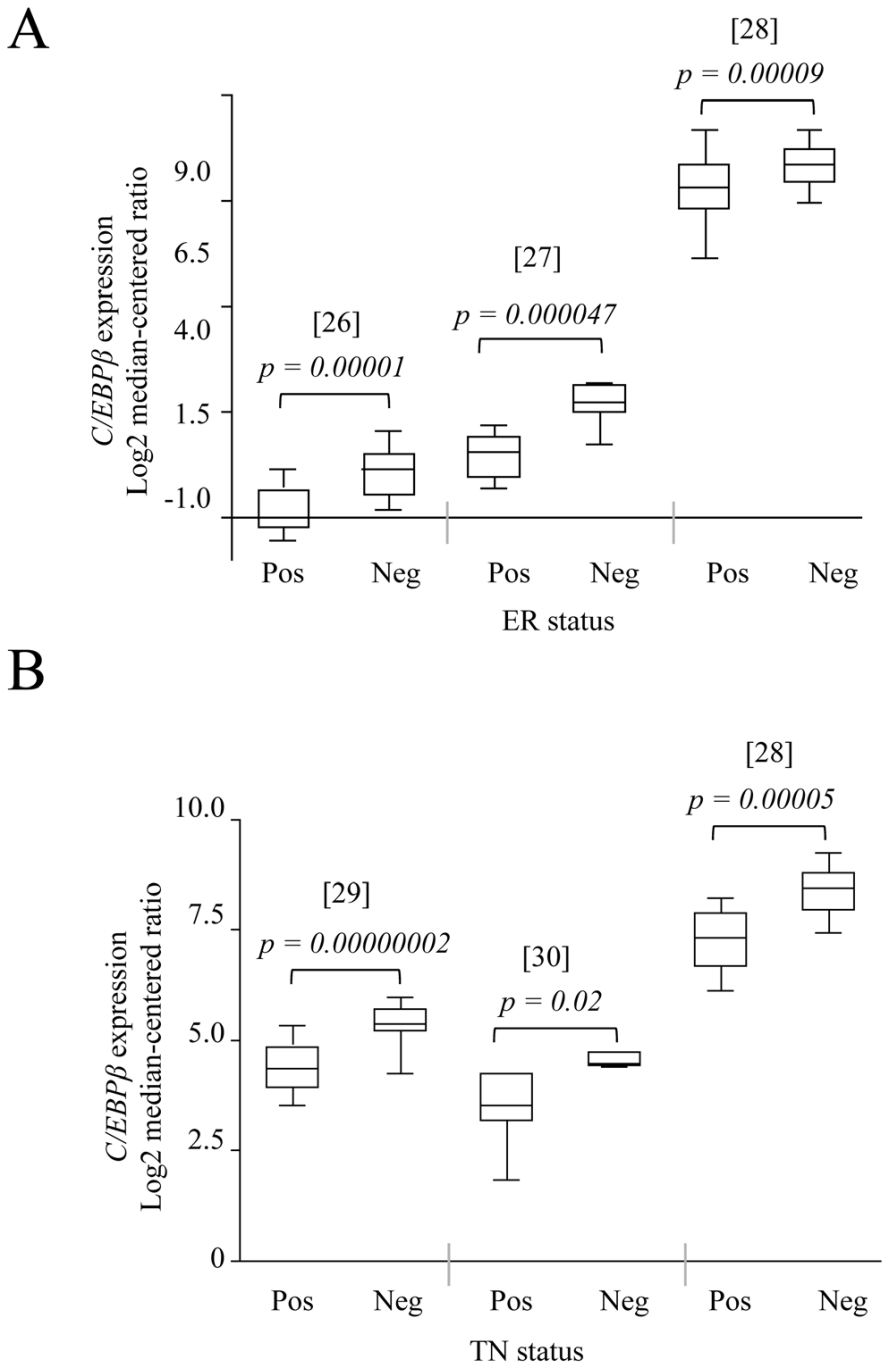
High C/EBPβ expression predicts poor outcome in human breast cancer. Data obtained from the Oncomine cancer microarray database (www.oncomine.org) showing higher C/EBPβ expression in (**A**) estrogen receptor (ER)-negative and (**B**) triple negative (TN) human breast carcinomas.

To further examine whether we could find an association between C/EBPβ and galectin-7 expression profiles in epithelial tissues, we looked at the Human Protein Atlas database, which contains high-resolution images showing the spatial distribution of proteins in normal and cancer tissues. We paid a particular attention to epithelial tissues known to express galectin-7 constitutively, including skin, esophagus, oral mucosa and cervical tissues. As expected, immunohistochemistry staining of normal epithelial tissues showed a predominantly nuclear pattern of C/EBPβ protein expression while galectin-7 was mostly found in the cytosolic and nuclear compartments (**Figure 4**). In all cases, the distribution of galectin-7 expression co-located with that of C/EBPβ. Identical findings were observed in normal mammary tissues where galectin-7 and C/EBPβ were both specifically expressed in myoepithelial cells. Using the ONCOMINE public cancer microarray database, we also found that C/EBPβ was expressed at significantly higher levels in oestrogen receptor (ER)-negative and triple-negative (TN) breast cancer tissues (**Figure 5**), a pattern identical to that of galectin-7 in human breast cancer tissues [9].

## Discussion

*Galectin-7* has generally been considered a gene under the control of p53. There is an increasing number of reports, however, showing that galectin-7 is overly expressed in cancer cells, most notably in as esophageal, lung and buccal squamous cell carcinomas, thyroid carcinomas, bladder cancer, lymphoma, and breast cancer [7], [20]–[25]. This is somewhat paradoxical since p53 is often inhibited by mutations within its DNA binding domain that lead to the expression of a transcriptionally inactive p53. Here, we have provided a possible explanation for this paradox. More specifically, we found that increased expression of C/EBPβ-2 is sufficient to upregulate galectin-7 expression at both mRNA and protein levels in breast cancer cells. Such a role for C/EBPβ was specific to C/EBPβ-2 since C/EBPβ-3, another isoform that often acts as a dominant negative factor against C/EBPβ-2, could not induce galectin-7 nor did it repress the constitutive expression of *galectin-7* or the C/EBPβ-2-induced galectin-7 expression. The role of C/EBPβ-2 as an important transcription factor for galectin-7 is further supported by our mutational analysis showing that mutation in the C/EBP binding motif located at position −147/−132 bp of the *galectin-7* promoter region strongly inhibits its transcriptional activity. It is also supported by data found in public databases, which showed that C/EBPβ and galectin-7 have an almost identical distribution pattern in both normal and cancer tissues. Taken together, these results identify a novel regulatory pathway that regulates *galectin-7* expression in human breast cancer cells.

Close examination of a number of public databases supports the view that C/EBP is a positive regulator of *galectin-7* in breast cancer cells. For example, a significant increase in *C/EBPβ* mRNA has been observed in estrogen and progesterone-receptor-negative breast cancer versus those tumors positive for these receptors [26]–[28]. An increase of *C/EBPβ* mRNA also correlates with metastatic breast cancer and high tumor grade, making it an interesting biomarker for subsets of tumors with a poor prognosis [28]–[30]. Moreover, our preliminary results have shown that transfection of C/EBPβ-2 in MCF-7 cells increases their motility (**Figure S1**). Similar results were obtained when we increased *galectin-7* expression (**Figure S2**), consistent with our previous findings that high levels of galectin-7 increase metastasis of breast cancer cells to the bone and the lung [9]. In fact, our results may have implications in other types of cancer where gal-7 is expressed, most notably in transformed keratinocytes. Both gal-7 and C/EBPβ-2 have been shown to be involved in the differentiation of keratinocytes [31]. Whether suppression of C/EBPβ-2 will necessarily reduces gal-7 is currently unknown but likely since previous studies showing that retinoic acid, which suppresses the expression of C/EBPβ-2 target genes in keratinocytes [32]–[33], also inhibits gal-7 expression [34]. Interestingly, C/EBPβ-2 has been shown to be expressed at high levels in most mammary tumors and is the most frequent isoform found in human breast cancer cell lines [35]. Its expression in MCF10A cells has also been shown to induce *in vitro* epithelial to mesenchymal transition associated with increased invasive properties [17]. However, while C/EBPβ-2 is generally considered the most transcriptionally active of all three C/EBPβ isoforms, we cannot completely rule out the implication of other isoforms in regulating gal-7 expression. The C/EBPβ-3 isoform, for instance, is expressed at high levels in some breast carcinoma, most notably in basal-like breast cancer [36]–[37]. Future studies with isoform-specific antibodies will thus be needed to correlate the expression of gal-7 with specific C/EBPβ isoforms in breast cancer tissues. It is important to note, however, that other transcription factors can also upregulate gal-7 and may thus compensate for suppression of C/EBPβ-2. This is particularly true for breast cancer cells which often express a mutant form of p53, which is capable of inducing gal-7 [38]. Future investigations will thus be needed to determine whether direct suppression of galectin-7 expression or via specific targeting C/EBPβ are valuable alternatives to inhibit breast cancer progression.

Our *in silico* analysis of the human *galectin-7* promoter has revealed some other interesting features, most notably within the C/EBP binding site located at position −147/−132 bp. This site contains an overlapping consensus binding motif for NF-κB. Overlapping binding motifs for transcription factors are frequently found genome-wide in both eukaryotic and prokaryotic cis-regulatory regions of a gene promoter. Such overlap often results in competitive binding of transcription factors to the overlapping site [20]. Our preliminary investigations on the relevance of the overlap in the consensus binding motifs for C/EBP and NF-κB at position −147/−132 bp have shown that a mutation (Δ-147-145) in the NF-κB recognition sequence of the *galectin-7* reporter construct that leaves intact the C/EBP recognition site increased rather than reduced the activity of the promoter (**Figure S3**). This would suggest that endogenous NF-κB units, such as transcriptionally inactive p50 complexes bound to this site, hinder binding C/EBP for the same site. This possibility is supported by our results showing that caffeic acid phenethyl ester (CAPE), which prevents NF-κB binding to DNA, increased C/EBPβ-2-induced *galectin-7* expression (**Figure S3**). The ability of C/EBPβ to displace NF-κB on the *galectin-7* promoter was confirmed by ChIP analysis which showed that while endogenous p50 is constitutively bound to the *galectin-7* promoter, transfection of C/EBPβ-2 displaced the p50 homodimers, leading to a strong activation of *galectin-7* expression. However, in presence of transcriptionally active NF-κB complexes, containing c-Rel for example, NF-κB could possibly exert a positive influence of galectin-7 expression, as we recently showed [38]. In other words, galectin-7 expression in cancer cells with an inactive p53 pathway possibly involves C/EBP and/or NF-κB. These observations also consistent with a model where expression of *galectin-7* gene is repressed by transcriptionally inactive p50:p50 homodimers that bind to this overlapping C/EBPβ/NF-κB site on *galectin-7* promoter. During malignant transformation, increased expression of C/EBPβ-2 or a transcriptionally active NF-κB complex displaces the p50 homodimers, leading to a strong activation of *galectin-7* expression. Future investigations will be needed to determine how specific signaling pathways can dictate an NF-κB or C/EBPβ response.

## Supporting Information

Figure S1 **Overexpression of C/EBPβ-2 enhanced migration of MCF-7 cells line.** Migration in 6-well plates of MCF-7 cells line transfected with pCMV5 vector (CTRL), C/EBPβ-2 or C/EBPβ-3 cDNA vectors. Plots of 30 cells/sample tracked by live cell imaging are represented. Quantifications of velocity, directionality, accumulated distance and euclidean distance represent mean values ± SEM from all plots.
(TIFF)

Figure S2 **Overexpression of galectin-7 enhanced migration of MCF-7 cells line.** Migration in 6-well plates of MCF-7 cells line transfected with Srα or galectin-7 cDNA vectors. Plots of 60 cells/sample tracked by live cell imaging are represented. Quantifications of velocity, directionality, accumulated distance and euclidean distance represent mean values ± SEM from all plots.
(TIFF)

Figure S3 **Binding of C/EBPβ and NF-κB on** ***galectin-7*** **promoter.** (A) Transcriptional activity of the *galectin-7* promoter following a mutation (Δ-147-145) that disrupts the NF-κB binding site located at −148 bp of a luciferase vector encoding a promoter construct of the human *galectin-7* gene. Transfection efficiency was normalized by co-transfection with a β-galactosidase reporter vector. (B) Effect of CAPE on C/EBPβ-2-induced *galectin-7* expression in MCF-7 cells. Expression was measured by semi-quantitative RT-PCR. GAPDH was used as loading control. (C) ChIP analyses showing displacement of the p50 complexes by C/EBPβ-2. Comparative binding of p50 complexes and C/EBPβ on the endogenous *galectin-7* promoter in control cells MCF-7 and cells transfected with an expression vector encoding C/EBPβ-2. Binding to the promoter was measured using anti-p50 and anti-C/EBPβ using genomic DNA. An isotypic antibody (IgG) was used as a negative control. Total DNA extract was used as a positive control (input).
(TIFF)
